# Comparative proteomic profiling of soleus, extensor digitorum longus, flexor digitorum brevis and interosseus muscles from the mdx mouse model of Duchenne muscular dystrophy

**DOI:** 10.3892/ijmm.2013.1429

**Published:** 2013-07-03

**Authors:** STEVEN CARBERRY, HEINRICH BRINKMEIER, YAXIN ZHANG, CLAUDIA K. WINKLER, KAY OHLENDIECK

**Affiliations:** 1 Department of Biology, National University of Ireland, Maynooth, Co. Kildare, Ireland; 2 Department of Pathophysiology, University of Greifswald, Greifswald, Germany

**Keywords:** dystrophinopathy, muscle dystrophic x-linked, muscular dystrophy, interosseus muscle, flexor digitorum brevis muscle

## Abstract

Duchenne muscular dystrophy is due to genetic abnormalities in the dystrophin gene and represents one of the most frequent genetic childhood diseases. In the X-linked muscular dystrophy (mdx) mouse model of dystrophinopathy, different subtypes of skeletal muscles are affected to a varying degree albeit the same single base substitution within exon 23 of the dystrophin gene. Thus, to determine potential muscle subtype-specific differences in secondary alterations due to a deficiency in dystrophin, in this study, we carried out a comparative histological and proteomic survey of mdx muscles. We intentionally included the skeletal muscles that are often used for studying the pathomechanism of muscular dystrophy. Histological examinations revealed a significantly higher degree of central nucleation in the soleus and extensor digitorum longus muscles compared with the flexor digitorum brevis and interosseus muscles. Muscular hypertrophy of 20–25% was likewise only observed in the soleus and extensor digitorum longus muscles from mdx mice, but not in the flexor digitorum brevis and interosseus muscles. For proteomic analysis, muscle protein extracts were separated by fluorescence two-dimensional (2D) gel electrophoresis. Proteins with a significant change in their expression were identified by mass spectrometry. Proteomic profiling established an altered abundance of 24, 17, 19 and 5 protein species in the dystrophin-deficient soleus, extensor digitorum longus, flexor digitorum brevis and interosseus muscle, respectively. The key proteomic findings were verified by immunoblot analysis. The identified proteins are involved in the contraction-relaxation cycle, metabolite transport, muscle metabolism and the cellular stress response. Thus, histological and proteomic profiling of muscle subtypes from mdx mice indicated that distinct skeletal muscles are differentially affected by the loss of the membrane cytoskeletal protein, dystrophin. Varying degrees of perturbed protein expression patterns in the muscle subtypes from mdx mice may be due to dissimilar downstream events, including differences in muscle structure or compensatory mechanisms that counteract pathophysiological processes. The interosseus muscle from mdx mice possibly represents a naturally protected phenotype.

## Introduction

Duchenne muscular dystrophy is the most frequent gender- specific genetic disease of the neuromuscular system and is characterised by symmetric and highly progressive muscle wasting. Degeneration of muscle fibers and subsequent fibrosis result in gait disturbance in early childhood followed by the loss of ambulation and severe cardiorespiratory complications in older children (1). Primary genetic abnormalities in the dystrophin gene cause the loss of a crucial membrane cytoskeletal protein of 427 kDa that is normally located in the subsarcolemmal region of the muscle fibers (2). Although considerable progress has been made in the development of genetic and cell-mediated approaches to eliminate the primary abnormality in dystrophinopathies (3), there is still the urgent need to address secondary alterations in the highly complex pathology of X-linked muscular dystrophy (4,5). One promising approach is the detailed molecular and cellular analysis of genetic animal models of muscular dystrophy and the subsequent evaluation of novel substances or treatment strategies in experimental phenotypes (6). In this respect, the X-linked muscular dystrophy (mdx) mouse is the most widely used animal model of Duchenne muscular dystrophy for studying secondary effects due to the reduction of the dystrophin-glycoprotein complex (7–10).

A point mutation in exon 23 induces a loss in the expression of the dystrophin isoform, Dp427, in muscle tissue from mdx mice (11). However, the same primary abnormality does not cause identical downstream effects in different muscles, making the mdx mouse an extremely interesting model system for studying secondary effects in dystrophinopathy. As previously reported, whilst the extraocular and laryngeal muscles are only mildly affected (12–14), the hind limb muscles exhibit a moderate dystrophic phenotype (15–17) and the diaphragm muscle is severely degenerated (18–20). The comparative analysis of differentially affected muscles may thus be helpful in determining how a single mutation in a muscle-specific gene can result in such a variety of pathophysiological phenotypes.

Over the last few years, mass spectrometry (MS)-based proteomics has been successfully applied to investigate normal and pathologically altered skeletal muscle tissue (21–23), establishing a variety of novel proteomic biomarkers of neuromuscular disorders (24). This has included the large-scale proteomic profiling of various muscle tissues from mdx mice and has revealed differential degrees of perturbed protein expression patterns in dystrophin-deficient fibers (25). Gel electrophoresis-based proteomics has been applied to evaluate dystrophic muscles from mdx mice focusing on the cytosolic fraction from 1- to 6-month-old hind limb muscle covering an isoelectric point (p*I*) range of 4–7 and using Coomassie and silver staining (26,27), crude extracts from gastrocnemius muscle from 9-week-old hind limb tissue covering a p*I* range of 3–10 and using Stains-All labelling (28), crude extracts from 9-week-old diaphragm tissue covering a p*I* range of 3–10 and using hot Coomassie staining (29), crude extracts from 9-week-old diaphragm tissue covering a p*I* range of 3–10 and using fluorescence two-dimensional difference in gel electrophoresis (2D-DIGE) (30), crude extracts from 10-week-old antisense oligomer-treated diaphragm tissue covering a p*I* range of 3–10 and using 2D-DIGE (31), extracts from 6-week-old gastrocnemius muscle covering a p*I* range of 3–10 and using 2D-DIGE (32), total extracts from 22-month-old diaphragm covering a p*I* range of 3–10 and using fluorescence labelling (33), total extracts from 22-month-old tibialis anterior muscle covering a p*I* range of 3–10 and using fluorescence labelling (34) and crude extracts from 9-week-old extraocular muscle covering a p*I* range of 3–10 and using fluorescence 2D-DIGE (35). Recently, gel-free *in vivo* SILAC proteomics was carried out with 3-week-old gastrocnemius muscle (36). In addition, proteomics has been used to evaluate novel protein factors in serum (37,38) and dystrophic heart tissue (39–42).

It is to be hoped that the establishment of a detailed biomarker signature of X-linked muscular dystrophy will improve our understanding of the pathobiochemical processes underlying dystrophinopathy. A comprehensive list of secondary effects would also be extremely useful for the biochemical evaluation of experimental treatments, such as stem cell therapy or exon-skipping approaches to ameliorate downstream alterations of dystrophin deficiency (4,5). The underlying objective of this study was the formation of an mdx reference map of differentially affected muscles with the same primary abnormality but diverging downstream effects. In humans, the equivalent mouse muscle tissue subtypes investigated relate to i) soleus (SOL), a muscle of the back part of the lower leg lying just beneath the gastrocnemius that contains almost exclusively oxidative fibers; ii) extensor digitorum longus (EDL), a muscle located in the lateral part of the front of the leg that contains a large portion of glycolytic type 2B and 2X fibers; iii) flexor digitorum brevis (FDB), a muscle that lies in the middle of the sole of the foot and contains a high number of oxidative-glycolytic type 2A fibers; and iv) interosseus (INT), muscles of the hand located near the metacarpal bones that contain predominantly type 2 fibers (43–46). All four muscles have been widely used to investigate alterations of muscle structure, function, physiology and biochemistry in mdx mice.

However, with respect to routine biological analyses, each of these muscle subtypes has strengths and weaknesses. While EDL and SOL are well suited to study muscle biochemistry, force and fatigue, FDB and INT can be enzymatically dissociated into single fibers. The two latter muscles can quite easily be studied on the single fiber level. Thus, many observations on Ca^2+^ influx, subsarcolemmal Ca^2+^ levels and the function of cation channels have been made with INT (9,47,48) and FDB (47,49,50) muscles without taking into consideration whether these findings can be generalised. Accordingly, we included FDB and INT muscle preparations in our proteomic analyses to evaluate whether they display similar abnormalities as compared to other limb muscles from mdx mice. Proteomics revealed an altered expression for 24, 17, 19 and 5 protein species in the dystrophic SOL, EDL, FDB and INT muscles, respectively. These marked differences in the degree of protein perturbation are possibly a result of dissimilar secondary processes in the molecular pathogenesis of different subtypes of muscles from mdx mice.

## Materials and methods

### Materials

Immobilised pH gradient strips of pH 3–10, IPG buffers and electrophoresis-grade chemicals were obtained from Amersham Biosciences/GE Healthcare (Little Chalfont, UK). Acrylamide stock solutions were purchased as ultrapure protogel mixtures from National Diagnostics (Atlanta, GA, USA). Fluorescent ruthenium II bathophenathroline disulfonate for the production of RuBPs dye was from Reagecon Diagnostics Ltd. (Shannon, Ireland). Coomassie blue dye, Laemmli-type gel buffers and protein molecular mass standards, as well as Bradford reagent for protein quantification were from Bio-Rad Laboratories (Hemel-Hempstead, UK). Chemiluminescence substrate and protease inhibitors were purchased from Roche Diagnostics (Mannheim, Germany). For the reproducible generation of peptide populations from 2D spots by in-gel digestion, sequencing grade-modified trypsin was obtained from Promega Corp. (Madison, WI, USA). LC-MS CHROMASOLV^®^ water and formic acid were from Fluka (Dorset, UK). Nitrocellulose transfer stacks were obtained from Invitrogen Life Technologies (Carlsbad, CA, USA). X-ray film was from Fuji Photo Film (Tokyo, Japan). Antibodies were purchased from Abcam (Cambridge, UK; ab77232 to myoglobin, ab9465 to actinin, ab11427 to parvalbumin, ab6588 to collagen, ab75223 to phosphoglycerate kinase, ab14226 to serpina and ab13496 to αB-crystallin) and Sigma (Dorset, UK; L9393 to laminin). All secondary antibodies were from Chemicon International (Temecula, CA, USA). Ponceau S red staining solution, DNase-I and all general chemicals were obtained from Sigma.

### Genetic mouse model of Duchenne muscular dystrophy

In analogy to patients suffering from X-linked muscular dystrophy, the mutant mdx mouse is missing the Dp427 isoform of the membrane cytoskeletal protein, dystrophin (8), and exhibits a drastic reduction in dystrophin-associated glycoproteins (51). The genetic basis of this abnormality is a single base substitution within exon 23 of the dystrophin gene (11). SOL, EDL, FDB and INT muscles from 3-month-old mdx mice (C57BL/10 ScSn Dmdy (mdx)/J) and age-matched wild-type (WT) mice (C57BL/10 ScSn) were obtained from the BioResource Unit of the University of Greifswald, Greifswald, Germany (52). The mouse strains were originally obtained from the Jackson Laboratory (Bar Harbor, ME, USA). Mice were kept under standard conditions and all procedures were performed in accordance with the German guidelines on the use of animals for scientific experiments (approval by District Veterinary Office, Anklam, Germany). The animals were sacrificed by cervical dislocation and muscle tissues were immediately removed. The samples used for proteomic analysis were quick-frozen in liquid nitrogen and those for histological staining were frozen in petroleum ether at −90°C.

### Histological Analyses

Frozen muscle tissue was cut into 7 μm cross sections using a cryostat (Frigocut 2800N, Leica). The sections were stained with hematoxylin and eosin (H&E) according to standard protocols. Central and peripheral nuclei of muscle fibers were counted. For evaluation, digital microscopic recordings with a magnification of ×200, were analysed. Muscles specimens from six mice per strain were investigated.

### Proteomic analysis

For the proteomic analysis of mdx tissue, muscle specimens were shipped to Ireland on dry ice and stored at −80°C prior to usage. In order to obtain sufficient protein extracts, 30 dystrophic and 30 normal specimens from each skeletal muscle investigated were pulverised by grinding the tissue pieces in liquid nitrogen using a mortar and pestle. The ground muscle powder was solubilised in lysis buffer with the ratio of 100 mg wet weight/ml lysis buffer [7 M urea, 2 M thiourea, 4% CHAPS, 2% IPG buffer pH 3–10, 2% (w/v) DTT]. To prevent excess protein degradation, the lysis buffer was supplemented with a freshly prepared protease inhibitor cocktail as previously descrbied (29). Following gentle rocking for 60 min, the suspensions were centrifuged at 4°C for 20 min at 15,000 × g (35) and the protein concentration was determined as previously described (53).

### 2D gel electrophoresis

The high-resolution 2D gel electrophoretic separation of the urea-soluble protein complement from normal vs. dystrophic SOL, EDL, FDB and INT muscle tissue was carried out using a total protein amount of 500 μg per analytical slab gel. Using a reswelling tray from Amersham Biosciences/GE Healthcare, IPG strips of pH 3–10 were rehydrated for 12 h with 0.45 ml of a rehydration buffer containing 7 M urea, 2 M thiourea, 65 mM CHAPS, 10 mg/ml DTT, and 500 μg of muscle protein sample, as well as 1% (v/v) ampholytes pH 3–10. As a tracking dye, the buffer was complemented with 0.05% (w/v) bromophenol blue. Following placement of the first-dimension strips (24 cm in length) gel-side up onto the Ettan IPGphor manifold and coverage with 108 ml of dry-strip cover fluid, gels were run on the IPGphor IEF system with the following isoelectric focusing running conditions: 80 V for 4 h, 100 V for 2 h, 500 V for 1.5 h, 1,000 V for 1 h, 2,000 V for 1 h, 4,000 V for 1 h, 6,000 V for 2 h, 8,000 V for 2.5 h, and 500 V holding step; and 8,000 V for 1 h if strips had been at the 500 V holding step. Gel strips were then equilibrated for 30 min; using during the first 15 min of equilibration a buffer system with 100 mM dithiothreitol and during the last 15 min of incubation an equilibration buffer containing 0.25 M iodoacetamide. The second dimension separation was carried out with an Ettan DALTtwelve system from Amersham Biosciences/GE Healthcare using standard 12.5% (w/v) slab gels. Following washing in sodium dodecyl sulfate-containing running buffer, isoelectric focusing strips were placed on top of second dimension gels and held in place with a 1% (w/v) agarose sealing gel. The comparative proteomic profiling of SOL, EDL, FDB and INT muscles required the employment of 32 slab gels, which were run at 0.5 W/gel for 60 min and then 15 W/gel was used until the blue dye front had disappeared from the bottom of the gel.

### Post-electrophoretic protein labelling with fluorescent ruthenium II tris(bathophenanthroline disulfonate) (RuBPs) dye

The staining of 2D gels was carried out with the fluorescent dye, RuBPs, as previously described (54). A stock solution of RuBPs dye was prepared as outlined by Rabilloud *et al*(55). Following fixation for 30 min in 30% ethanol and 10% acetic acid, the gels were washed three times for 30 min in 20% ethanol and then stained for 6 h in 20% (v/v) ethanol containing 2 μM of ruthenium chelate. The gels were re-equilibrated twice for 10 min in distilled water and destained overnight in 40% ethanol and 10% acetic acid prior to imaging (41). Fluorescently labelled proteins were visualised using a Typhoon Trio variable mode imager (Amersham Biosciences/GE Healthcare). Gel analysis was performed with Progenesis SameSpots analysis software from Nonlinear Dynamics (Newcastle upon Tyne, UK) using the following parameters: ANOVA p<0.05; n=4; and a power value >0.8. The 12.5% (w/v) slab gels used in this study separated muscle-associated proteins ranging in molecular mass from approximately 15–220 kDa. Proteins in spots with a significant increase or decrease in abundance (differing between the various groups with >2-fold change) were identified by MS.

### MS identification of muscle proteins

The mass spectrometric identification of proteins of interest was carried out with 2D protein spots from Coomassie-stained pick gels, following counter-staining of RuBPs-labelled analytical gels. Standardised in-gel tryptic digestion was used for the generation of representative peptide mixtures (54). The excision of gel plugs, washing and destaining of protein spots and treatment with trypsin were performed by a previously optimised method (56,57). Trypsin-generated peptides were harvested by removing supernatants from digested gel plugs after centrifugation. Further recovery was achieved by the addition of 30% acetonitrile/0.2% trifluoroacetic acid to the gel plugs for 10 min at 37°C with gentle agitation. The resulting supernatants were pooled with the initially recovered peptides following trypsin digestion (58). Peptide samples were dried through vacuum centrifugation and concentrated fractions were suspended in MS-grade distilled water and 0.1% formic acid, spun down through spin filters and added to LC-MS vials for identification by ion trap LC-MS analysis. Electrospray ionization LC-MS/MS analysis was carried out as previously described (56) using a Model 6340 Ion Trap/LC-MS apparatus from Agilent Technologies (Santa Clara, CA, USA). The separation of peptides was performed with a nanoflow Agilent 1200 series system equipped with a Zorbax 300SB-C18 analytical reversed phase column using HPLC-Chip technology. Mobile phases used were A, 0.1% formic acid; B, 50% acetonitrile and 0.1% formic acid. Samples were loaded into the enrichment part of the chip at a capillary flow rate set to 4 μl/min with a mixture of solvent A and solvent B at a ratio of 19:1. Tryptic digests were eluted with a linear gradient of 5–70% solvent B over 6 min, 70–100% solvent B over 1 min, 100–5% over 1 min (56). A 5-min post-time of solvent A was used to remove any potential carry-over. The capillary voltage was set to 2,000 V. The flow and temperature of the drying gas were 4 l/min and 300°C, respectively. Database searches were carried out with Mascot MS/MS ion search (Matrix Science Ltd., London, UK; NCBI database, release 20100212). All searches used ‘*Mus musculus*’ as taxonomic category and the following parameters: i) two missed cleavages by trypsin; ii) mass tolerance of precursor ions ±2.5 kDa and product ions ±0.7 kDa; iii) carboxymethylated cysteins fixed modification; iv) oxidation of methionine as variable modification; and v) at least two matched distinct peptides. Mascot scores >50 are listed in Tables I–IV. All p*I*-values and molecular masses of identified proteins were compared to the relative position of their corresponding 2D-spots on analytical slab gels.

### Verification of key proteomic findings by immunoblot analysis

In order to verify potential alterations in the concentration of select muscle-associated proteins and to confirm the findings from the comparative proteomic profiling of dystrophic muscle specimens from mdx mice, immunoblotting of proteins of interest was carried out. Following the electrophoretic transfer of proteins onto nitrocellulose membranes, the sheets were blocked in a fat-free milk protein solution for 1 h and then incubated overnight with gentle agitation with primary antibody, sufficiently diluted in blocking solution containing 5% (w/v) milk powder in phosphate buffered saline [PBS; 0.9% (w/v) NaCl, 50 mM sodium phosphate, pH 7.4] as previously described (56). Following washing with blocking solution twice for 10 min, the blots were incubated for 1 h with secondary peroxidase-conjugated antibodies, diluted in blocking solution. Antibody-decorated bands in washed blots were visualised by the enhanced chemiluminescence method following the manufacturer’s recommendations. Densitometric scanning of immunoblots was performed on a Computing Densitometer 300S (Molecular Dynamics, Sunnyvale, CA, USA) with ImageQuant tools V3.0 software (29).

## Results

### Histological profiling of skeletal muscle tissue from mdx mice

**Figure 1:**
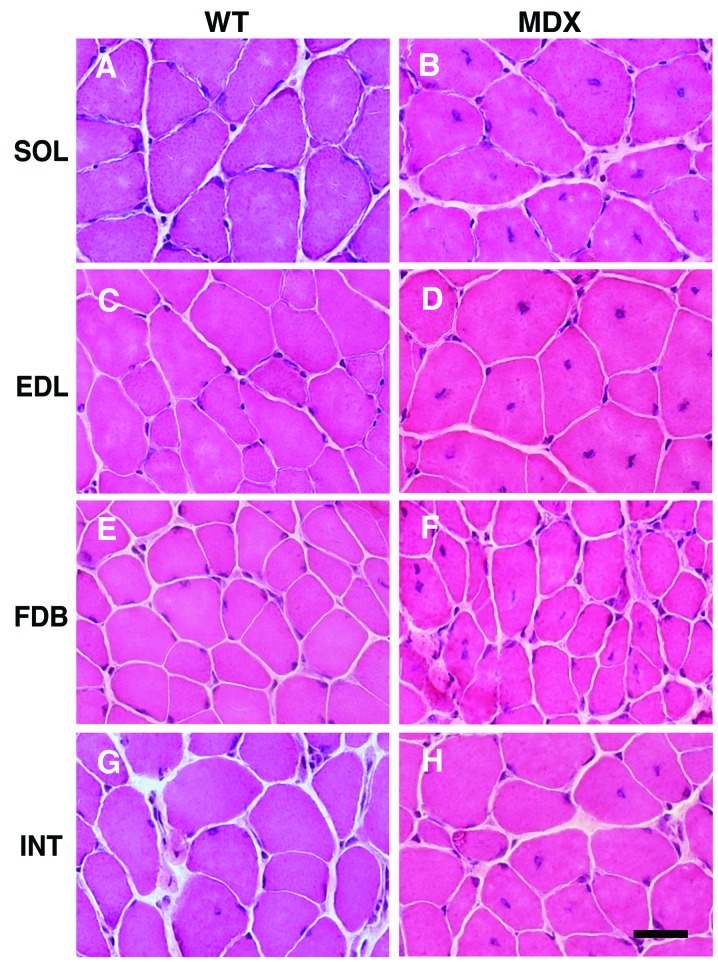
Histological profiling of skeletal muscles from X-linked muscular dystrophy (mdx) mice. Shown are hematoxylin and eosin-stained muscle cross-sections from wild-type (WT; A, C, E and G) and mdx mice (B, D, F and H). Note the reduced occurrence of central nuclei in the flexor digitorum brevis (FDB) and *interosseu*s (INT) muscles, compared with the soleus (SOL) muscle and extensor digitorum longus (EDL) muscle from mdx mice. Muscles were prepared from 100-day-old male animals. Scale bar, 20 μm.

**Figure 2:**
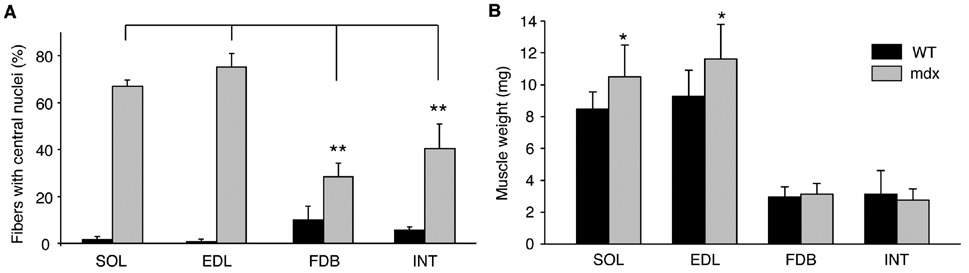
Degree of central nucleation and muscle weight of different mdx skeletal muscles. (A) Shown is the graphical presentation of the degree of central nucleation in wild-type (WT) vs. dystrophic soleus (SOL), extensor digitorum longus (EDL), flexor digitorum brevis (FDB) and interosseus (INT) muscles from X-linked muscular dystrophy (mdx) mice. The fractions of fibers with central nuclei are presented as a percentage (%) for the four muscles (mdx. n=6; WT, n=4 independent muscles and animals). In each muscle cross-section approximately 100 fibers were counted and classified. Data from the FDB and INT muscles from mdx mice were tested for significant differences to those of the SOL and EDL muscles. Statistical tests were performed independently for all pairs. (B) The average muscle weight is provided for the four muscles (n=4–6). (A and B) The means ± SD are provided in all cases; asterisks (*) indicate significant differences (Mann-Whitney U test; ^*^p<0.05; ^**^p<0.01).

The histological examination of cross sections from the SOL, EDL, FDB and INT muscles revealed central nucleation in all tested types of skeletal muscle from mdx mice (Fig. 1B, D, F and H). In addition, the muscles revealed fiber size variations and the occurrence of small, rounded fibers. As expected, central nuclei were rarely observed in the muscle fibers from WT mice (Fig. 1A, C, E and G). Quantitative evaluation of central nucleation revealed a significantly lower degree of nucleation in the FDB and INT muscles compared with the SOL and EDL muscles (Fig. 2A). It is well known that dystrophin-deficient muscle can develop a mild hypertrophy, at least at certain stages. While the body weight of mdx mice was almost identical to that of the WT mice (data not shown), the mass of the EDL and SOL muscles from the mdx animals was on average 20–25% higher. By contrast, no evidence of hypertrophy was observerd for the two smaller muscles, FDB and INT (Fig. 2B).

### Comparative proteomic profiling of skeletal muscle tissue from mdx mice

**Figure 3:**
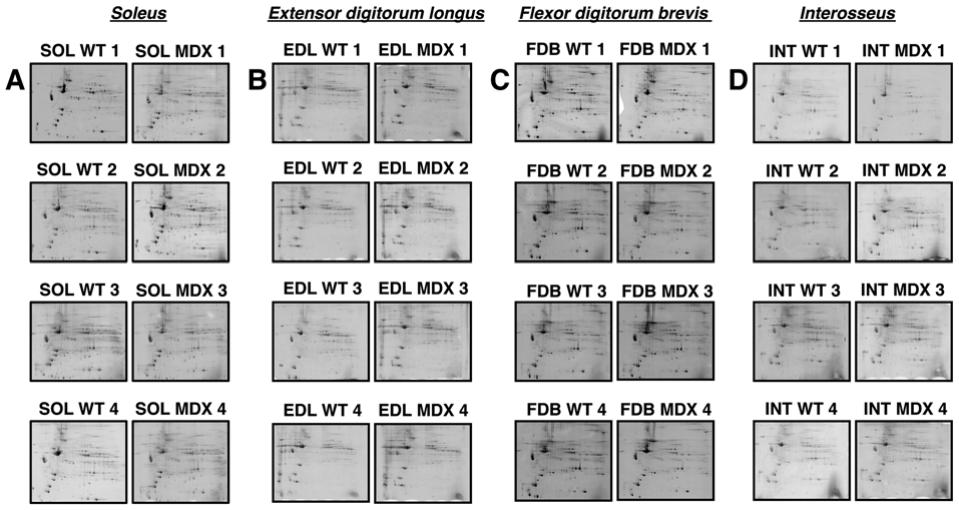
Overview of two-dimensional (2D) gel electrophoretic survey of skeletal muscles from X-linked muscular dystrophy (mdx) mice. Shown areRuBPs-stained gels of total extracts from 3-month-old normal wild-type (WT) vs. dystrophic (MDX) (A) soleus (SOL), (B) extensor digitorum longus (EDL), (C) flexor digitorum brevis (FDB) and (D) interosseus (INT) muscles. The 32 gel images contain four biological repeats of each group of normal (wild-type; WT 1–4) vs. diseased (MDX 1–4) muscles. Fluorescent images are shown for the pH 3–10 range.

**Figure 4:**
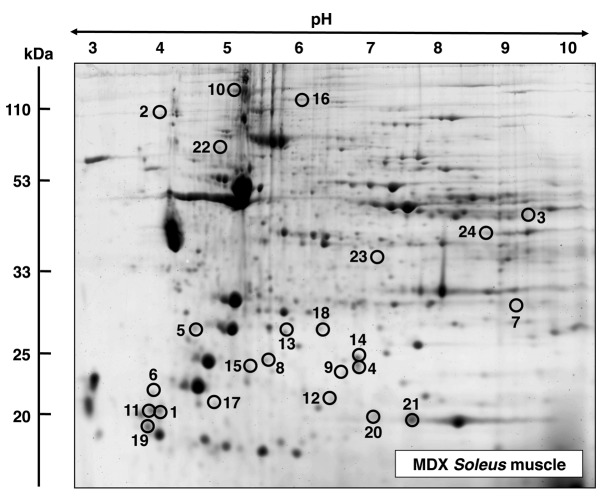
Fluorescence two-dimensional (2D) gel electrophoretic analysis of soleus muscle from mdx mice. Shown is a representative RuBPs-labelled master gel of crude tissue extracts from soleus muscle from mdx mice. Protein spots with an age-related change in expression levels are marked by circles and are numbered 1–24. See Table I for the mass spectrometric identification of individual muscle-associated proteins. The pH-values of the first dimension gel system and molecular mass standards of the second dimension are indicated on the top and on the left of the panels, respectively.

**Figure 5:**
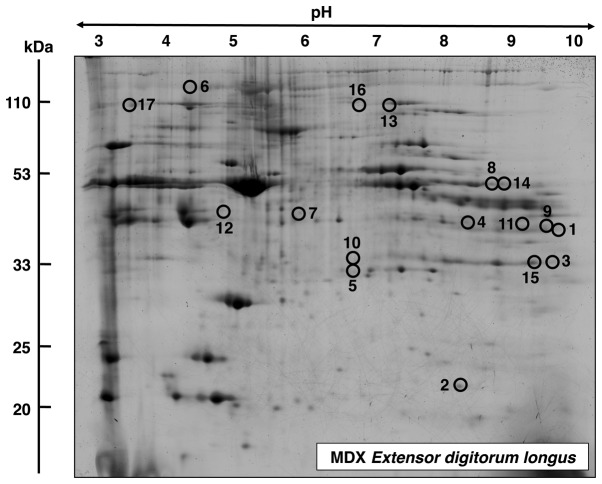
Fluorescence two-dimensional (2D) gel electrophoretic analysis of extensor digitorum longus muscle from mdx mice. Shown is a representative RuBPs-labelled master gel of crude tissue extracts from extensor digitorum longus muscle from mdx mice. Protein spots with an age-related change in expression levels are marked by circles and are numbered 1–17. See Table II for the mass spectrometric identification of individual muscle-associated proteins. The pH-values of the first dimension gel system and molecular mass standards of the second dimension are indicated on the top and on the left of the panels, respectively.

**Figure 6:**
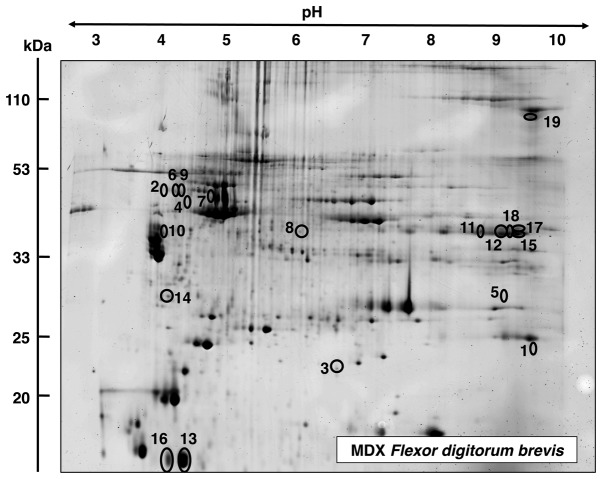
Fluorescence two-dimensional (2D) gel electrophoretic analysis of flexor digitorum brevis muscle from mdx mice. Shown is a representative RuBPs-labelled master gel of crude tissue extracts from dystrophic flexor digitorum brevis muscle. Protein spots with an age-related change in expression levels are marked by circles and are numbered 1–19. See Table III for the mass spectrometric identification of individual muscle-associated proteins. The pH-values of the first dimension gel system and molecular mass standards of the second dimension are indicated on the top and on the left of the panels, respectively.

**Figure 7:**
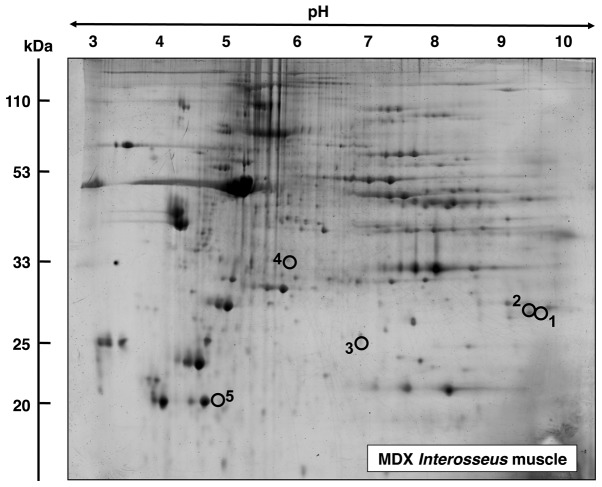
Fluorescence two-dimensional (2D) gel electrophoretic analysis of interosseus muscle from mdx mice. Shown is a representative RuBPs-labelled master gel of crude tissue extracts from dystrophic interosseus muscle. Protein spots with an age-related change in expression levels are marked by circles and are numbered 1–5. See Table IV for the mass spectrometric identification of individual muscle-associated proteins. The pH-values of the first dimension gel system and molecular mass standards of the second dimension are indicated on the top and on the left of the panels, respectively.

High-resolution 2D gel electrophoresis was used to separate the urea-soluble portion of the assessable proteome from normal vs. dystrophic SOL, EDL, FDB and INT muscle tissues. Post-electrophoretic labelling of 2D protein spots was achieved with the fluorescent dye, RuBPs, and protein species with a significantly altered abundance in dystrophic preparations were determined by densitometric scanning. MS was then employed to identify the muscle-associated proteins of interest. Fig. 3 summarizes analytical gels used to evaluate whether marked differences exist in the degree of protein perturbation between different subtypes of dystrophin-deficient skeletal muscles. Individual panels A–D show four biological repeats of gel electrophoretic analyses of normal (SOL WT 1–4) vs. dystrophic (SOL MDX 1–4) SOL, normal (EDL WT 1–4) vs. dystrophic (EDL MDX 1–4) EDL, normal (FDB WT 1–4) vs. dystrophic (FDB MDX 1–4) FDB and normal (INT WT 1–4) vs. dystrophic (INT MDX 1–4) INT muscle tissues, respectively. Since the overall 2D protein spot patterns of WT vs. dystrophic specimens (MDX) were relatively comparable, a detailed densitometric analysis for the determination of significant differences in muscle-associated proteins was carried out. With the help of a Typhoon Trio variable imager and Progenesis 2D analysis software, skeletal muscle proteomes from mdx mice were compared. Figs. 4–7 represent master gels of the SOL, EDL, FDB and INT muscles, respectively.

### Proteomic analysis of SOL muscle from mdx mice

A fluorescent RuBPs-labelled master gel of mdx SOL muscle is shown in Fig. 4. An altered expression was observed for 24 protein species in the dystrophin-deficient SOL preparations. Proteins with significant changes in expression levels are marked by circles and are numbered 1–24 in the master gel. The mass spectrometric identification of these muscle proteins with changes in expression is listed in Table I. This table includes the names of the identified muscle-associated proteins, their international accession number, p*I*-values, their relative molecular masses, number of matched peptide sequences, percentage sequence coverage, Mascot scores and fold change of individual proteins affected in the SOL muscle from mdx mice. Protein species with an altered expression in the SOL muscle ranged in molecular mass from 17 kDa (myoglobin) to 127 kDa (myosin binding protein) and covered a p*I*-range from p*I* 4.7 (myosin light chain) to 8.9 (malate dehydrogenase). As listed in Fig. 4 and Table I, proteomic profiling suggests a dystrophy-associated increase in various myosin light chains (MLCs), including MLC1/3, MLC2 and MLC3 (spots 1, 5, 11, 17 and 19), cadherin (spot 2), aldolase (spot 3), αB-crystallin (spots 4, 9 and 14), troponin C (TnC) (spot 6), glutathione transferase (spot 7), 14-3-3 protein (spots 8 and 15), collagen (spot 10) phosphatase (spot 12), ferritin (spot 13), slow myosin binding protein C (MyBP-C) (spot 16) and peroxiredoxin (spot 18). By contrast, decreased concentrations were observed in the case of tumor metastatic process-associated protein NM23 (spot 20), myoglobin (spot 21), the ATP synthase Atp5b (spot 22), creatine kinase (spot 23) and malate dehydrogenase (spot 24).

### Proteomic analysis of EDL muscle from mdx mice

A master gel of dystrophic EDL muscle is presented in Fig. 5, illustrating an altered expression in 17 protein species. The mass spectrometric identification of these muscle-associated proteins is listed in Table II. Proteins with an altered density in EDL muscle ranged in molecular mass from 17 kDa (myoglobin) to 224 kDa (myosin) and covered a p*I*-range from p*I* 5.2 (actin) to p*I* 9.0 [troponin T (TnT)]. MS-based proteomics revealed an increase in fast troponin TnT (spots 1 and 9), myoglobin (spot 2), phosphoglycerate mutase (spots 3 and 15), glyceraldehyde-3-phosphate dehydrogenase (spot 4), triosephosphate isomerase (spot 5), myosin (spot 6), lactate dehydrogenase (spot 7), phosphoglycerate kinase (spot 8), creatine kinase (spot 10), malate dehydrogenase (spot 11), actin (spot 12), glycogen phosphorylase (spot 13) and phosphoglycerate kinase (spot 14). A decreased abundance was established for glycogen phosphorylase (spot 16) and actinin (spot 17).

### Proteomic analysis of FDB muscle from mdx mice

The proteomic survey of dystrophic FDB muscle is shown in Fig 6. Proteins with an altered expression are marked in the master gel and numbered 1–19. Table III lists the mass spectrometric identification of these muscle-associated proteins. Proteins ranged in molecular mass from 12 kDa (parvalbumin) to 130 kDa (collagen) and covered a p*I*-range from p*I* 4.9 (vimentin) to 9.2 (collagen). Mass spectrometric analyses revealed an increased concentration in FDB muscle for fast troponin I (TpI) (spot 1), serpina 1d protein (spots 2, 6 and 9), αB-crystallin (spot 3), vimentin (spot 4), phosphoglycerate mutase (spot 5), desmin (spot 7), leukocyte elastase inhibitor A (spot 8) and tropomyosin (spot 10). Proteins with a decreased abundance in FDB muscle were shown to be the glycolytic enzyme aldolase (spots 11, 12, 15, 17 and 18), parvalbumin (spots 13 and 16), 14-3-3 protein (spot 14) and collagen (spot 19).

### Proteomic analysis of INT muscle from mdx mice

In contrast to the above listed findings of considerable changes in the proteomes from dystrophic SOL, EDL and FDB muscle, the mass spectrometric analysis of INT muscle revealed only a limited number of altered proteins. In the master gel shown in Fig. 7, proteins with a change in expression are numbered 1–5. Table IV lists the proteomic identification and fold change of these proteins in dystrophic INT muscle. An increased expression level in INT muscle was shown for fast TpI (spots 1 and 2), the molecular chaperone αB-crystallin (spot 3) and the 40 kDa protein (spot 4). By contrast, the cytosolic Ca^2+^-binding protein parvalbumin was found to be decreased in the INT muscle.

### Immunoblot analysis of mdx muscle preparations

**Figure 8:**
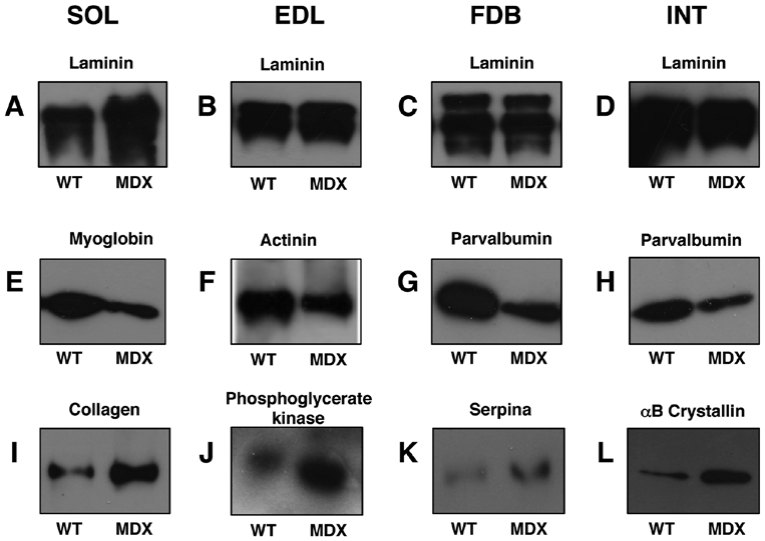
Immunoblot analysis of normal vs. dystrophic skeletal muscle. Shown are representative immunoblots with expanded views of antibody-decorated protein bands. Lanes 1 and 2, 3 and 4, 5 and 6 and 7 and 8 represent normal wild-type (WT) vs. dystrophic (MDX) preparations from soleus (SOL; A, E and I), extensor digitorum longus (EDL; B, F and J), flexor digitorium brevis (FDB; C, G and K) and interosseus (INT; D, H and L) muscles, respectively. Blots were labelled with antibodies to (A–D) laminin, (E) myoglobin, (F) actinin, (G and H) parvalbumin, (I) collagen, (J) phosphoglycerate kinase, (K) serpina and (L) αB-crystallin.

In order to verify key proteomic findings, comparative immunoblot analysis was carried out. As shown in Fig. 8, the altered abundance of two marker proteins from each analysed muscle was confirmed by antibody labelling. Panels A–D were stained with an antibody to laminin for control purposes. The extracellular matrix protein exhibited relatively comparable amounts in normal vs. dystrophic specimens, with the exception of the SOL muscle which showed an increased density in the mdx preparations. The reduced concentration of myoglobin and increased concentration of collagen in the SOL muscle (Fig. 8E and I), the decreased levels of actinin and increased concentration of phosphoglycerate kinase in EDL muscle (Fig. 8F and J), the lower levels of parvalbumin and higher levels of serpina in the FDB muscle (Fig. 8G and K) and the reduced expression of parvalbumin and increased concentration of αB-crystallin in the INT muscle (Fig. 8H and L) was verified by immunoblot analysis.

## Discussion

The proteomic survey of four subtypes of skeletal muscle, i.e., SOL, EDL, FDB and INT muscle, presented in this study revealed considerable differences in the extent of protein perturbations in distinct muscles of the mdx model of Duchenne muscular dystrophy. While 24, 17 and 19 protein species were altered in the SOL, EDL and FDB muscles from mdx mice, respectively, dystrophin-deficient INT muscle preparations showed only alterations in fast TnI, αB-crystallin, the 40 kDa protein and parvalbumin. This is an interesting finding from a global analysis of protein expression patterns in dystrophinopathy and agrees with the idea that the loss of dystrophin and concomitant reduction of associated glycoproteins results in considerably different secondary changes and cellular adaptations in individual skeletal muscles, despite the fact that all contractile mdx tissues exhibit the same primary genetic abnormality. Two accompanying observations on INT muscle from mdx mice agree with the proteomic analyses. Firstly, the number of fibers containing central nuclei was lower in the INT compared with the SOL and EDL muscles. This is crucial, since central nucleation is regarded as a reliable indicator of recent muscle fiber regeneration. Secondly, INT and FDB muscles from mdx mice did not show any increase in muscle mass, in contrast to the SOL and EDL muscles. Hypertrophy of dystrophin-deficient muscles has been observed in some models of Duchenne muscular dystrophy, including the mdx mouse (59). However, the signaling pathways causing muscle growth have not yet been fully elucidated (60,61). Nevertheless, both central nucleation and the degree of hypertrophy are in line with a less severe impairment of INT, compared with the SOL and EDL muscles from mdx mice. Thus, results on the pathophysiology of dystrophin-deficient muscle should be carefully interpreted if they are merely obtained with INT fibers from mdx mice (9,47).

Previous proteomic studies of mdx muscle tissues concur that the deficiency in the full-length Dp427 isoform of the membrane cytoskeletal protein, dystrophin, results in disturbed protein expression patterns in contractile tissues (26–36). Although earlier studies on mdx muscle proteomics differ considerably on the listing of individual proteins involved in the molecular pathogenesis of muscular dystrophy (25), it is clear that the disintegration of sarcolemmal integrity has severe consequences for the overall function of affected muscle fibers. Interestingly, severe dystrophic diaphragm muscle exhibits extensive alterations in the expression of a large number of muscle proteins (29–31,33), while mildly affected dystrophic extraocular muscle shows much less alterations in its proteome (35). This demonstrates a correlation between the pathophysiological phenotype of individual muscles from mdx mice and the degree of proteome-wide changes. For example, extraocular muscle fibers appear to be naturally protected, possibly due to the upregulation of the autosomal dystrophin-homologue, utrophin Up395 (12). In addition, different load-bearing capacities and Ca^2+^-extrusion systems may render certain subtypes of skeletal muscle less susceptible to fibrosis and necrosis, despite the lack of dystrophin (12–14,62).

Muscle-associated proteins with an altered abundance in mdx tissues are mostly involved in the contraction-relaxation cycle, metabolite transport, muscle metabolism and the cellular stress response. In the mildly affected dystrophic INT muscle, the protein with the most significant increase in expression was fast TpI (63), indicating a certain degree of remodeling of the regulatory elements of the contractile apparatus (64). Other changes in protein expression in the dystrophic INT muscle were marginal, compared with the other muscles examined. In the dystrophic SOL muscle, various MLCs, such as isoforms MLC1/3, MLC2 and MLC3 (65), were shown to be upregulated in the dystrophin-deficient fibers. The highly complex myosin molecule of the contractile apparatus forms a hexameric structure consisting of two MHC heavy chains and various MLC light chains (66,67). Various combinations of myosin heavy and light chains form a plethora of fiber type-specific isoforms (68) and the MS-based profiling of contractile fibers has shown that the actomyosin apparatus is extremely plastic (69). Previous proteomic investigations have demonstrated that neuromuscular activity has a profound effect on myosin, actin, troponin and tropomyosin isoform expression patterns (57,70). The drastic changes in the dystrophic SOL muscle indicate that the dystrophic phenotype is associated with considerable remodeling of the contractile apparatus, including myosin, myosin binding proteins and troponin.

Interestingly, the atypical GPI-anchored cadherin 13 protein is increased in dystrophic SOL muscle and may stimulate angiogenesis (71). Altered expression levels of glutathione S-transferase, αB-crystallin, ferritin and peroxiredoxin indicate increased demands of detoxification, cellular stress response, iron storage and anti-oxidant activity in dystrophic fibers. Of note, specific isoforms of 14-3-3 proteins are altered in muscular dystrophy, which has been previously described for various neurodegenerative processes (72). Possible interactions of 14-3-3 proteins with signaling receptors, kinases and phosphatase are disturbed in dystrophic SOL muscle. Reduced levels of ATP synthase, myoglobin and malate dehydrogenase indicate metabolic disturbances in dystrophic SOL muscle.

Apart from altered levels of contractile elements, such as troponin, myosin, actin and actinin in dystrophic EDL muscle, which demonstrated alterations in the actomyosin apparatus, proteomic analysis revealed a striking increase in the expression levels of key glycolytic enzymes. MS identified the affected cytosolic proteins as triosephosphate isomerase, glyceraldehyde-3-phosphate dehydrogenase, phosphoglycerate kinase and phosphoglycerate mutase, which are involved in the reversible conversion of dihydroxyacetone phosphate and glyceraldehyde-3-phosphate, the generation of 1,3-bisphosphoglycerate and NADH, the production of ATP and 3-phosphoglycerate from ADP and 1,3-bisphosphoglycerate, and the reversible conversion of 3-phosphoglycerate into 2-phosphoglycerate, respectively (73). The increase in several glycolytic enzymes suggests a shift to more anaerobic metabolism. Of note, one of the key regulatory enzymes of glycogen and glucose utilization in muscle, glycogen phosphorylase, was also found to be increased in contractile mdx tissue. A previous proteomic survey of slow-twitching vs. fast-twitching skeletal muscles has confirmed that fast muscles exhibit high concentrations of enzymes involved in the glycolytic pathway and elevated levels of glycogen phosphorylase (74). In addition, the elevated expression of lactate dehydrogenase, an enzyme that mediates the interconversion of the end product of glycolysis, pyruvate, and lactate agrees with a glycolytic shift in dystrophic EDL muscle. Skeletal muscles utilise anaerobic glycolysis, usually for short to moderate duration activities of high intensity (75); thus, a significant increase in glycolytic enzymes indicates an increased utilization of the glycolytic pathway in the bioenergetics of the dystrophin-deficient EDL muscle.

In contrast to dystrophic EDL muscle, the dystrophic FDB muscle exhibited a reduced expression in a key glycolytic enzyme. The affected enzyme, skeletal muscle aldolase, mediates the reversible biochemical breakdown of fructose-1,6-biphosphate into dihydroxyacetone phosphate and glyceraldehyde-3-phosphate (73). In the gluconeogenic pathway, muscle aldolase has been shown to form a complex with fructose-1,6-biphosphatase and α-actinin on both sides of the Z-line of skeletal muscle fibers. The tight association between fructose-1,6-biphosphatase and aldolase is proposed to enable efficient substrate channeling between these proteins (76). However, many glycolytic enzymes are believed to have a multi-functional role in many cell types (77). Thus, changes in glycolytic enzymes may affect biological mechanisms other than the anaerobic breakdown of glucose in skeletal muscle tissues (73). For example, enzymes with a primary glycolytic function have been demonstrated to also being involved in the regulation of apoptosis, metabolic integration, stimulation of cell motility and transcriptional regulation (77). It is thus difficult to conclude from changes in one glycolytic enzyme whether this relates to distinct metabolic alterations in the dystrophic FDB muscle or possibly shows adaptations in a glycolysis-unrelated biological mechanism.

Altered expression levels in troponin and tropomyosin, and the cytosolic Ca^2+^-binding protein parvalbumin indicate disturbances in the contractile apparatus and ion homeostasis, respectively. In analogy to dystrophic SOL muscle, the apparent upregulation of the molecular chaperone αB-crystallin in dystrophic FDB agrees with the concept of an enhanced cellular stress response in muscular dystrophy (30). Interestingly, cytoskeletal proteins, such as vimentin and desmin were found to be increased in dystrophic muscle, which may be due to a compensatory mechanism of dystrophin-lacking fibers that try to counteract structural instabilities in the membrane cytoskeleton of dystrophic FDB muscle. The increased levels of the serpina 1d protein may be the basis of a novel biomarker of the dystrophic phenotype.

In conclusion, the comparative proteomic survey of four frequently used muscles of the mdx mouse model of Duchenne muscular dystrophy, SOL, EDL, FDB and INT muscles, has demonstrated that high-resolution 2D gel electrophoresis in combination with electrospray ionization MS is highly suitable for investigating muscle subtype-specific alterations in the dystrophic skeletal muscle proteome. The differences in the number and degree of protein alterations in the analysed mdx muscles indicate that the INT muscle is much less affected than SOL, EDL and FDB muscles. In agreement with these findings are the lack of hypertrophy in INT muscles of mdx mice and the reduced level of central nucleation compared with SOL and EDL. Thus, the evaluation of future pharmacological studies or experimental gene therapeutic approaches for the treatment of dystrophinopathy should take into account that the deficiency in dystrophin does not affect all skeletal muscle subtypes in a similar manner. The individual histological, biochemical and physiological properties of different muscles have to be considered for the understanding of secondary abnormalities and adaptations in muscular dystrophy.

## Figures and Tables

**Table I:** List of muscle-associated proteins with an altered abundance in the soleus muscle from mdx mice.

Spot no.	Protein name	Accession no.	Mascot score	p*I*	Molecular mass (Da)	Peptides matched	Sequence coverage (%)	Fold change
1	Myosin light chain 2 (MLC2)	AAA39796	401	4.71	18870	9	60	5.0
2	Cadherin 13	AAH21628	104	4.9	78474	2	4	4.4
3	Aldolase A, isoform 2	NP031464	136	8.31	39795	17	40	4.3
4	αB-crystallin	NP034094	121	6.76	20056	3	28	4.2
5	Myosin light chain 3 (MLC3)	EDL09001	268	5.03	22523	6	38	3.3
6	Troponin C, skeletal muscle	NP033420	190	4.07	18156	2	20	3.1
7	Glutathione S-transferase	NP034488	415	7.71	26069	9	42	2.8
8	14-3-3 Protein γ	NP036611	145	4.8	28519	4	9	2.8
9	αB-crystallin	NP034094	58	6.76	20056	1	14	2.8
10	Collagen α-1 (VI) chain	NP034063	224	5.2	109582	13	16	2.6
11	Myosin light chain 2 (MLC2)	NP058034	272	4.82	19059	4	31	2.5
12	Mg-dependent phosphatase 1	NP075886	98	6.29	18629	3	29	2.4
13	Ferritin light chain 1	P29391	372	5.66	20848	6	44	2.3
14	αB-crystallin	NP034094	141	6.76	20056	13	60	2.3
15	14-3-3 Protein ζ	BAA13421	258	4.7	27911	4	18	2.2
16	Myosin binding protein C, slow	HQ848554	411	5.74	127046	9	10	2.1
17	Myosin light chain 2 (MLC2)	NP058034.1	161	4.82	19059	5	44	2.1
18	Peroxiredoxin-1	NP035164	170	8.26	22394	5	24	2.0
19	Myosin light chain 1/3 (MLC1/3)	NP067260	514	4.98	20697	11	53	2.0
20	Tumor metastatic process-associated protein NM23	AAA39826	141	8.44	18846	4	40	−2.0
21	Myoglobin	NP038621	161	7.07	17117	2	21	−2.1
22	Atp5b protein	BC037127	488	5.24	56632	7	20	−2.2
23	Creatine kinase M-type	NP031736	97	6.58	43250	2	7	−2.3
24	Malate dehydrogenase	AAA39509	132	8.93	36052	5	22	−4.2

p*I*, isoelectric point.

**Table II:** List of muscle-associated proteins with an altered abundance in the extensor digitorum longus muscle from mdx mice.

Spot no.	Protein name	Accession no.	Mascot score	p*I*	Molecular mass (Da)	Peptides matched	Sequence coverage (%)	Fold change
1	Troponin T, fast skeletal muscle	AAB39743	66	9.01	29358	2	11	3.8
2	Myoglobin	NP038621	547	7.07	17117	11	68	3.7
3	Phosphoglycerate mutase2	NP061358	484	8.65	28983	11	49	3.4
4	Glyceraldehyde-3-phosphate dehydrogenase	AAH85315	347	7.59	36099	7	30	3.0
5	Triosephosphate isomerase	AAB48543	90	5.62	22724	4	22	2.6
6	Myosin-1	NP109604	774	5.60	224131	19	11	2.6
7	Lactate dehydrogenase B chain	NP032518	775	5.70	36839	15	49	2.5
8	Phosphoglycerate kinase	AAA70267	299	7.53	44914	6	21	2.3
9	Troponin T, fast skeletal muscle	AAB39743	131	9.01	29358	6	31	2.3
10	Creatine kinase M-type	NP031736	114	6.58	43250	5	16	2.2
11	Malate dehydrogenase	AAA39509	313	8.93	36052	11	41	2.1
12	Actin, α skeletal muscle	NP001091	136	5.23	42372	10	31	2.1
13	Glycogen phosphorylase	NP035354	1047	6.65	97689	25	35	2.0
14	Phosphoglycerate kinase	AAA70267	109	7.53	44914	7	22	2.0
15	Phosphoglycerate mutase 2	NP061358	637	8.65	28983	14	51	2.0
16	Glycogen phosphorylase	NP035354	54	6.65	97689	2	4	−2.6
17	α-actinin-3	NP038484	374	5.31	103616	7	9	−2.8

p*I*, isoelectric point.

**Table III:** List of muscle-associated proteins with an altered abundance in the flexor digitorum brevis muscle from mdx mice.

Spot no.	Protein name	Accession no.	Mascot score	p*I*	Molecular mass (Da)	Peptides matched	Sequence coverage (%)	Fold change
1	Troponin I, fast skeletal muscle	NP033431	57	8.65	21518	5	30	3.1
2	Serpina 1d protein	AAH21850	104	5.18	46140	9	24	2.7
3	αB-crystallin	NP034094	324	6.76	20056	8	45	2.4
4	Vimentin	CAA69019	261	4.96	51591	21	50	2.4
5	Phosphoglycerate mutase 2	NP061358	75	8.65	28983	2	8	2.3
6	Serpina 1d protein	AAH21850	136	5.18	46140	5	11	2.3
7	Desmin	NP034173	206	5.21	53523	10	32	2.2
8	Leukocyte elastase inhibitor A	NP079705	178	5.85	42722	9	30	2.1
9	Serpina 1d protein	AAH21850	131	5.18	46140	7	17	2.0
10	Tropomyosin, β chain	NP033442	176	4.66	32933	17	47	2.0
11	Aldolase A, isoform 2	NP001170778	202	8.31	39795	15	48	−2.2
12	Aldolase A, isoform 2	NP001170778	249	8.31	39795	13	40	−2.2
13	Parvalbumin α	NP038673	228	5.02	11923	8	65	−2.3
14	14-3-3 Protein γ	AAC14345	116	4.80	28519	8	38	−2.3
15	Aldolase A, isoform 2	NP001170778	163	8.31	39795	13	40	−2.7
16	Parvalbumin α	NP038673	219	5.02	11923	8	65	−3.0
17	Aldolase A, isoform 2	NP001170778	139	8.31	39795	9	27	−3.1
18	Aldolase A, isoform 2	NP001170778	215	8.31	39795	20	55	−3.3
19	Pro-α-2(I) collagen	CAA41205	51	9.23	130046	2	2	−3.4

p*I*, isoelectric point.

**Table IV:** List of muscle-associated proteins with an altered abundance in the interosseus muscle from mdx mice.

Spot no.	Protein name	Accession no.	Mascot score	p*I*	Molecular mass (Da)	Peptides matched	Sequence coverage (%)	Fold change
1	Troponin I, fast skeletal muscle	NP033431	301	8.65	21518	8	29	4.1
2	Troponin I, fast skeletal muscle	NP033431	245	8.65	21518	5	21	3.4
3	αB-crystallin	NP034094	421	6.76	20056	9	56	2.2
4	40 kDa Protein	1405340A	164	4.80	32848	3	15	2.1
5	Parvalbumin, α	NP038673	400	5.02	11923	7	63	−2.2

p*I*, isoelectric point.

## Abbreviations

EDL: extensor digitorum longus
FDB: flexor digitorum brevis
INT: interosseus
SOL: soleus
mdx: X-linked muscular dystrophy
2D: two-dimensional
RuBPs: ruthenium II tris(bathophenanthroline disulfonate)
